# Polymer Based Whispering Gallery Mode Humidity Sensor

**DOI:** 10.3390/s18072383

**Published:** 2018-07-22

**Authors:** Ann Britt Petermann, Thomas Hildebrandt, Uwe Morgner, Bernhard Wilhelm Roth, Merve Meinhardt-Wollweber

**Affiliations:** 1 Hannover Centre for Optical Technologies (HOT), Leibniz Universität Hannover, Nienburger Strasse 17, D-30167 Hannover, Germany; ann-britt.petermann@hot.uni-hannover.de (A.B.P.); thomas.hildebrandt@hot.uni-hannover.de (T.H.); bernhard.roth@hot.uni-hannover.de (B.W.R.); 2 Institute of Quantum Optics, Leibniz Universität Hannover, Welfengarten 1, D-30167 Hannover, Germany; morgner@iqo.uni-hannover.de

**Keywords:** polymer photonics, integrated optics device, humidity sensor

## Abstract

Whispering gallery mode (WGM) resonators are versatile high sensitivity sensors, but applications regularly suffer from elaborate and expensive manufacturing and read-out. We have realized a simple and inexpensive concept for an all-polymer WGM sensor. Here, we evaluate its performance for relative humidity measurements demonstrating a sensitivity of 47 pm/% RH. Our results show the sensor concepts’ promising potential for use in real-life applications and environments.

## 1. Introduction

Monitoring and measuring the relative humidity (RH) is an important factor in many chemical processes and industrial applications, for example in air quality management, food manufacturing or in metal processing and polymer synthesis [1]. There is a wide range of different possibilities for humidity detection based on electrical, as well as optical principles [2].

Optical techniques have the advantage of immunity against electrical and vibrational influences and offer interesting multiplexing capabilities, among others [3]. Optical humidity sensors are based on radiation losses in optical waveguides [4], fiber Bragg gratings (FBGs) [5,6] or whispering gallery modes (WGMs), for example [7]. When using a radiation loss-based sensor, it is difficult to separate the response of the sensor to humidity from changes in laser power and also varying losses in optical connectors. Silica-based FBGs need a humidity-sensitive polymer coating to be functional. This coating material swells in response to humidity and reduces the refractive index, leading to a change of the Bragg wavelength. Unfortunately, the swelling also causes bending stress in the fiber. No additional coating is necessary when polymer optical fiber Bragg gratings (POFBGs) are used, but the response time could be very slow (typical sensitivities are in the range of 33.5 pm/% RH) [8].

WGM-based sensors enable very long photon life times or high quality factors, respectively. This makes them ideal sensors for many applications, especially for biological and chemical sensing [9]. Typical WGM resonator geometries are spheres, rings or toroids. WGM resonators confine light at specific wavelengths, called resonance wavelengths. The electromagnetic field is located close under the surface of the resonator, leading to a strong evanescent field outside. The resonance wavelength λ depends on, among others, the effective refractive index $n_{eff}$ and the radius of the resonator *R*. Hence, changing one of these parameters will cause a resonance wavelength shift Δλ [10]:

(1) $$\frac{\Delta \lambda }{\lambda }=\frac{\Delta n_{eff}}{n_{eff}}+\frac{\Delta R}{R}.$$

$\Delta n_{eff}$ is the change in the effective refractive index and ΔR the change in the size of the resonator. Humidity sensors based on WGM resonators were successfully tested in the last few years. For example, Mehrabani et al. used a polymer-coated silica microtoroid and reached a sensitivity of 12.98 pm/% RH [1]. Bhola et al. instead used a sol-gel-clad microring resonator with a sensitivity of 16 pm/% RH [11]. The manufacturing process of these sensors is expensive and time consuming. Moreover, their handling requires precise adjustment of different components. Consequently, WGMs have not been used in routine humidity measurement yet, even though they have proven to be highly sensitive sensors for this application.

As a first step to overcome these issues, we developed an inexpensive, easy to handle, easy to manufacture and completely polymer-based sensor. The general setup of the WGM sensor concept was introduced earlier for the application of wavelength measurement [12]. Using polymer materials for the resonators makes an additional coating unnecessary [8]. In this article, we realize a polymer WGM-based humidity sensor achieving high sensitivity and characterize its performance.

## 2. Materials and Methods

### 2.1. Experimental Setup

**Figure 1:**
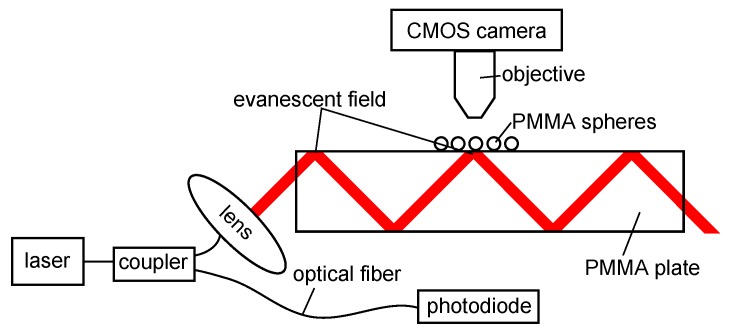
Experimental setup: Ten percent of the laser intensity is detected with a photodiode for calibration purposes. The remaining ninety percent of the laser intensity is collimated and coupled under 45° in a PMMA plate and guided based on total internal reflection. PMMA spheres supporting the whispering gallery modes (WGMs) are placed in the evanescent field present at the plate surface. The light distribution is captured by a CMOS camera equipped with a microscope objective.

As outlined in the Introduction, the sensor concept was developed to rely on a simple scheme and to be easy to manufacture. The design is shown in Figure 1. A PMMA plate with dimensions 50 mm × 50 mm × 2 mm forms the core of the sensor. The beam of a tunable narrow-band laser (TLB-6700, Newport Spectra-Physics GmbH, Darmstadt, Germany) with a tuning speed of 5 nm/s, a linewidth of ≤ 200 kHz and a resolution of 0.01 nm was collimated and coupled under 45° into this plate.

The plate acts as an optical waveguide due to total internal reflection. Thus, an evanescent field is present at the surface of the plate. In this field, commercially available PMMA spheres (Bang Laboratories, Fishers, IN, USA) were placed with a random distribution and positions. Two different mean diameters, 74.44 μm (all spheres smaller than 90 μm) and 165 μm (diameters between 150 μm and 180 μm), respectively, were used to investigate the influence of the resonator size. For a more flexible use and to ensure a more stable sensor configuration, the spheres could be fixed to the plate with a thin spin-coated layer of a UV curing adhesive (OG675, John P. Kummer GmbH, Augsburg, Germany) [13]. At a particular wavelength, only some of the spheres are in resonance, because the sphere diameters vary. Therefore, the total light distribution of all spheres changes with the incident wavelength. The generated intensity pattern of the spheres was captured by a CMOS camera (DCC1645C, Thorlabs, Newton, MA, USA) via a microscope objective (M-10X, Newport Spectra Physics GmbH, Darmstadt, Germany). Ten percent of the excitation light was detected with a photodiode (PDA36A-EC, Thorlabs, Newton, MA, USA) for intensity normalization.

In order to evaluate the sensor performance, the whole sensor was enclosed in an acrylic box, against environmental influences. To adjust the relative humidity, a Petri dish with a saturated salt solution was positioned inside the box together with the sensor [14]. By using lithium chloride (Carl Roth GmbH & Co. KG, Karlsruhe, Germany) at a temperature of 25.42 ${}^{\circ }C$, it is possible to modify the relative humidity inside the box between 25% and 40%, For the determination of the actual relative humidity, a commercially available relative humidity sensor (TSP01 and M00426120, Thorlabs, Newton, MA, USA) was placed in the box, as well. Our setup also contained a temperature sensor. The control of the temperature is crucial, because temperature changes also induce resonance wavelength shifts [12,15].

### 2.2. Characterization and Measurement Procedure

**Figure 2:**
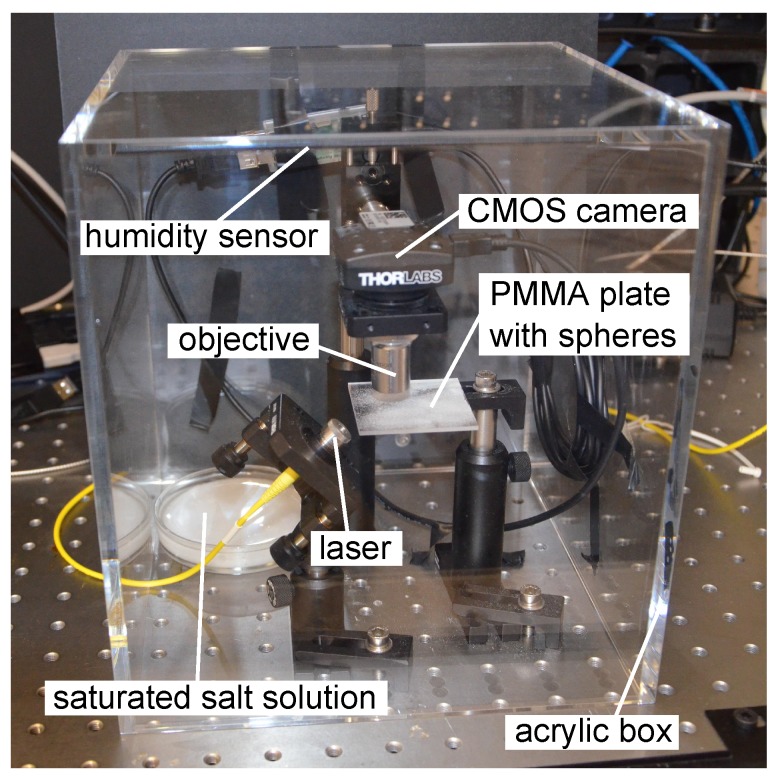
The WGM sensor, the commercially available relative humidity sensor and the saturated salt solution for humidity adjustment, placed under an acrylic box.

**Figure 3:**
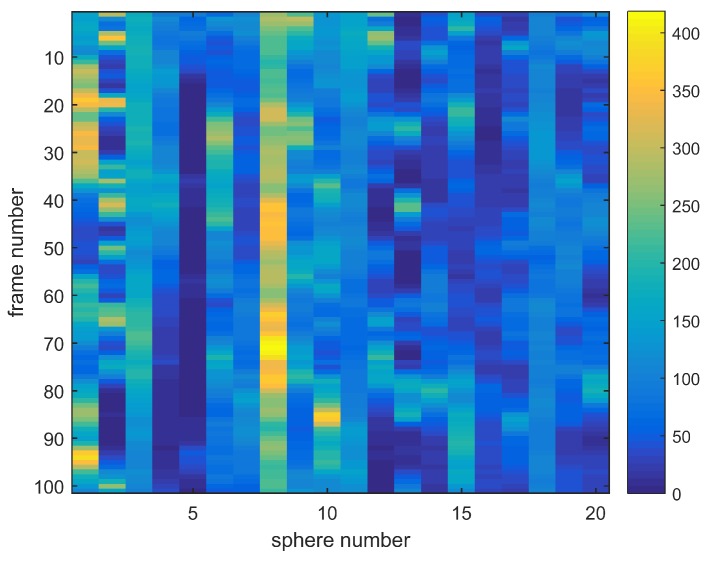
Mode map for an array of twenty spheres with a mean diameter of 74.44 μm (the frame number relates to wavelength).

Before the sensor can be used for relative humidity measurements [12,16], it needed to be calibrated once. In the calibration procedure, the exciting wavelength was scanned from 635 nm to 636 nm in 0.01 nm steps, and the intensity profile of the spheres at each wavelength was captured by the CMOS-camera (see Figure 2). Afterwards the positions and areas of the spheres that showed the strongest intensity changes were identified, and the associated pixel values were integrated over the sphere areas to obtain an integrated intensity for each sphere at each calibration wavelength. The laser power recorded with the photodiode is used to normalize the obtained intensity values. These intensity values for all spheres are stored together in a mode map. Figure 3 shows the mode map for an array of twenty spheres with a mean diameter of 74.44 μm. Due to small differences in the spheres’ diameters, only some spheres are in resonance at a specific wavelength marked as yellow bars in the mode map.

In each sphere, a variety of different modes is excited, so specific modes cannot be distinguished by the intensity measurements recorded with the sensor sketched in Figure 1. The approach here is that the intensity pattern of many spheres is unique, and therefore, an unambiguous determination of the present resonance condition is possible. To determine an unknown relative humidity, the wavelength was scanned again at different environmental conditions, and the resultant intensity values of the spheres for each specific wavelength were compared to the mode map measured in the first step via the correlation function r(λ) [12,16,17]:

(2) $$r\left(\lambda \right)=\sum_{j=1}^{N}\left|I_{j}^{DB}\left(\lambda \right)-I_{j}\right|.$$

**Figure 4:**
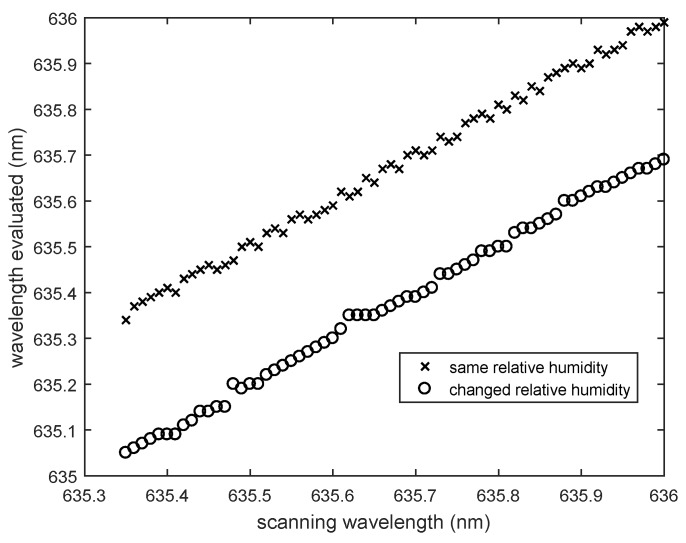
Comparison of scanning wavelengths and determined wavelengths, if the relative humidity changes (circles) or remains constant (crosses).

λ is the actual scanning wavelength; $I_{j}^{DB}$ is the intensity of the *j*-th sphere in the calibration mode map; and $I_{j}$ the intensity of this sphere at the unknown relative humidity. The correlation function has a minimum at that wavelength where the intensity profile at the unknown relative humidity fits the mode map best. In case the relative humidity does not change, the evaluated wavelength will be the same as the scanning wavelength. For example: the intensity profile of a sphere array at an unknown relative humidity at a scanning wavelength of 635.5 nm is compared to the calibration mode map. If the relative humidity has not changed, the correlation function has a minimum at 635.5 nm. If this is not the case, the relative humidity has changed, and the wavelength difference is proportional to the relative humidity variation. Figure 4 illustrates this dependence, showing a plot of the scanning wavelength versus the determined wavelength as obtained via the correlation function. For the curve with circles, the RH has changed, whereas for the curve with crosses, the RH remained constant.

## 3. Results

**Figure 5:**
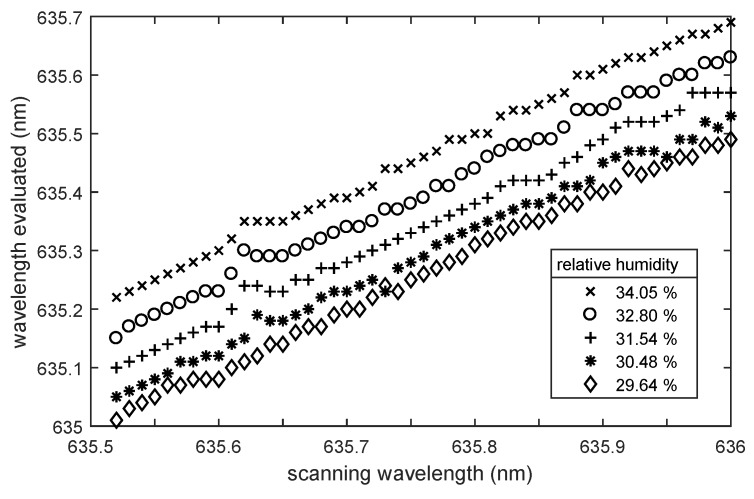
Determined wavelength compared to the laser scanning wavelength for different relative humidity levels. The initial relative humidity was 40.1%, and the array consisted of twenty 74 μm spheres.

Different variants of the sensor array were investigated to evaluate the performance of the polymer-based WGM sensor concept for measurement of relative humidity. Each tested array consisted of approximately twenty spheres either with a mean diameter of 74 μm or 165 μm, respectively. In addition, arrays with fixed 74 μm spheres were fabricated. As described in Section 2.1, samples were placed in an closed acrylic box, and the relative humidity inside the box was adjusted by a salt solution. As the relative humidity is expected to translate to a spectral shift in the resonance pattern of the array, we compared the true excitation wavelength to the wavelength measured by the array at different humidity conditions. The sensors’ behavior and sensitivity were characterized at different humidity levels by scanning the wavelength of the exciting laser, taking an image of the array under test at each wavelength and finally comparing these images to the related mode map of the array from the initial calibration. Figure 5 exemplarily shows the results for an array consisting of twenty 74 μm spheres without fixation.

**Figure 6:**
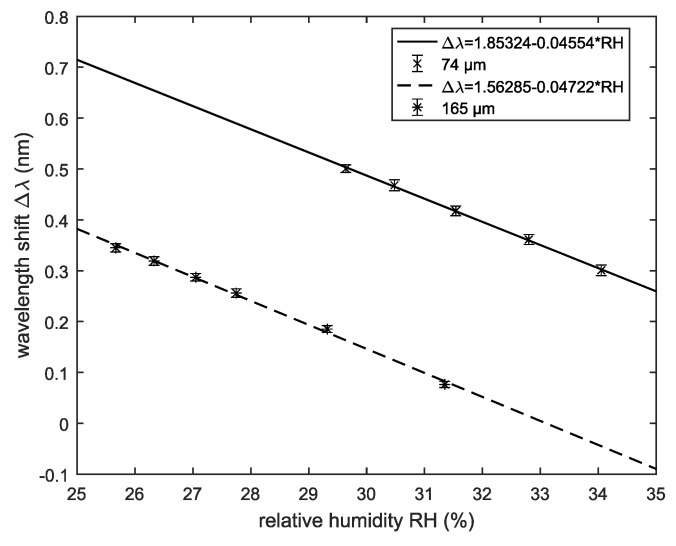
Wavelength shift Δλ as a function of relative humidity for arrays consisting of 74 μm spheres and 165 μm spheres, respectively. The slope of the curves is equal to the sensitivity.

The relative humidity used for initial calibration was 40.1%, With decreasing RH, the evaluated wavelength shifted to smaller values (blue shift). To determine the sensitivity, the wavelength differences between evaluated and scanning wavelength were calculated. This was done for both average sphere sizes utilized. Figure 6 shows the calculated wavelength shift Δλ as a function of the relative humidity (RH). The slope of the curves is equal to the related sensitivity.

The sensitivity for the 74 μm-sphere array is 0.046 nm/% RH. For the 165 μm-sphere array, 0.047 nm/% RH is obtained. Therefore, the sensitivity for the larger spheres is slightly bigger. Furthermore, the measurement error is very small. As shown in previous work, the accuracy could be improved by adding more spheres to the system [12]. Here, the arrays consist of twenty 74 μm and twenty-four 165 μm spheres, respectively, which is a large number compared to arrays used in previous works [12,13].

**Figure 7:**
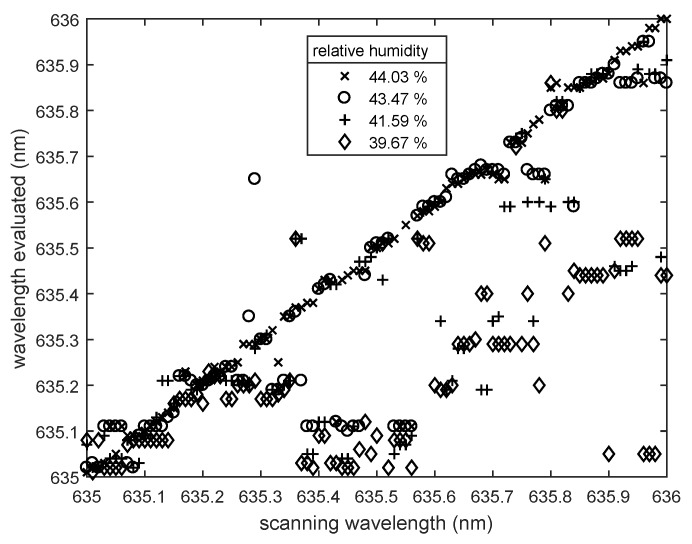
Determined wavelength compared to the laser scanning wavelength for a fixed sensor array at different relative humidity levels. The initial relative humidity was 44.46%, and the array consisted of twenty fixed spheres with diameter of 74 μm.

For a more flexible use of the sensor and for a better protection against environmental influences, the spheres can be fixed to the substrate. Previously, we showed that the sensitivity loss of a sensor with fixation is small against a sensor without fixation when determining an unknown wavelength [13]. To test if the sensor with fixed spheres is also a suitable relative humidity sensor, we used an array with fixed 74 μm spheres and repeated the procedure as described above. Figure 7 shows the results for an array with twenty spheres and a initial relative humidity of 44.46%.

First, despite decreasing RH, we observed that the evaluated wavelength remained at the true exciting wavelength. When the relative humidity change became larger, the evaluated wavelength differed from the actual exciting wavelength, but not in a systematic way, as observed before by the unfixed spheres. We repeated the test with different fixed arrays and obtained the same result. Obviously, the fixation layer was the reason for this. The diameter of the spheres varied in response to the changes of the relative humidity. A change of refractive index was also possible, but not expected to be large enough to explain the pronounced spectral shift of the resonance conditions. The fixation layer probably shielded a part of each sphere from the influence of the surrounding humidity. It may have also mechanically counteracted humidity-induced diameter changes. Therefore, the sphere diameters and consequently also the evaluated wavelength remained constant. If the relative humidity change became larger, the sphere diameters would change, but not in a well-determined way. As the fixed spheres started to deform, the mode profile of the deformed spheres was not comparable to the original mode profile stored in the mode map. A comparison of the wavelength-dependent resonance patterns was, thus, no longer possible, and the relative humidity could not be measured accurately.

## 4. Discussion and Conclusions

Today, humidity sensors utilizing whispering gallery mode resonators generally consist of only one resonator [1,11]. Consequently, high quality resonators are crucial for such a system. To achieve high quality factors, the resonator material is often glass [18], requiring a humidity-sensitive coating for application in humidity measurement [1,2,11].

In this work, we presented an all-polymer whispering gallery mode sensor concept and evaluated the performance of different design variants in humidity measurements. The design has two main advantages compared to the standard single-resonator design: firstly, using many spheres instead of one softens the high demands on resonator quality; and secondly, using PMMA instead of silica makes an additional humidity-sensitive coating unnecessary. As the requirements concerning the quality of the spheres are low in our design, commercially available spheres can be used, ensuring that the device is inexpensive and easy to manufacture.

The experimental evaluation of different sensor configurations (fixed and unfixed spheres, large and small spheres) for RH measurement showed promising results. For unfixed spheres, we achieved sensitivities of 47 pm/% RH for the 165 μm spheres and 46 pm/% RH for the 74 μm spheres, respectively. The achieved sensitivities are higher compared to those reported previously in the literature with other whispering gallery mode-based humidity sensors such as 12.98 pm/% RH for a polymer-clad silica microtoroid [1] or 16 pm/% RH for a polymer-clad microring resonator [11]). Rather, our results are comparable to those achieved with FBG-based humidity sensors (e.g., 33.6 pm/% RH [8]).

Even though the sensor was not able to reliably measure humidity in the fixed sphere configuration, more favorable results may be achieved with an improved fixation layer, e.g., reduced coverage of the sphere surface by the fixation material and/or using a more elastic material. In view of the transfer of the sensor concept to real-life applications, it needs to be mentioned that in the experiments presented here, the temperature was constant over the whole trial period. In a real environment, this will, rather, not be the case. As temperature changes induce diameter changes, as well, the resonance pattern of the spheres will shift accordingly. Consequently, the two effects need to be separated in a humidity measurement. This can be realized in the future by making one part of the sensor array insensitive to humidity, for example by application of a protective layer. With this protected part, it would be possible to measure temperature variations simultaneously with the same sensor array in order to correct the evaluated humidity response for the actual temperature.
